# Uptake of Digital Health Interventions for Cardiometabolic Disease in British South Asian Individuals: Think Aloud Study

**DOI:** 10.2196/57338

**Published:** 2024-10-24

**Authors:** Mel Ramasawmy, Dan Roland Persson, David Sunkersing, Paramjit Gill, Kamlesh Khunti, Lydia Poole, Wasim Hanif, Ann Blandford, Madiha Sajid, Fiona Stevenson, Nushrat Khan, Amitava Banerjee

**Affiliations:** 1 Institute of Health Informatics, University College London, 222 Euston Road, London, NW1 2DA, United Kingdom, 44 07940058826; 2 Department of Applied Mathematics and Computer Science, Wolfson Institute of Population Health, Queen Mary University of London, London, United Kingdom; 3 Technical University of Denmark, Kongens Lyngby, Denmark; 4 Warwick Medical School, University of Warwick, Coventry, United Kingdom; 5 Diabetes Research Centre, Leicester General Hospital, University of Leicester, Leicester, United Kingdom; 6 Department of Psychological Interventions, School of Psychology, University of Surrey, Guildford, United Kingdom; 7 Department of Diabetes, University Hospital Birmingham, Birmingham, United Kingdom; 8 University College London Interaction Centre, University College London, London, United Kingdom; 9 Patient and Public Involvement Representative, Digital Interventions for South Asians with Cardiometabolic Disease Study, London, United Kingdom; 10 Department of Primary Care and Population Health, University College London, London, United Kingdom; 11 Department of Primary Care and Public Health, Imperial College London, London, United Kingdom; 12 Department of Cardiology, Barts Health National Health Service Trust, London, United Kingdom

**Keywords:** digital health, cardiometabolic disease, cardiology, cardiovascular risk, health inequality, health disparity, usability, user experience, think aloud, cultural barriers, digital divide, digital literacy

## Abstract

**Background:**

Digital health interventions (DHIs) could support prevention and management of cardiometabolic disease. However, those who may benefit most often experience barriers to awareness and adoption of these interventions.

**Objective:**

Among South Asian individuals, we evaluated user experience of DHIs for prevention and management of cardiometabolic disease, aiming to understand barriers and facilitators to initial and ongoing use.

**Methods:**

Among South Asian individuals recruited via primary care, community organizations, and snowball methods (n=18), we conducted “think-aloud” interviews using a reflective and reactive approach. Participants included nonusers, as well as those that used a range of DHIs as part of monitoring and improving their health. Participants were asked to think aloud while completing a task they routinely do in a familiar DHI, as well as while setting up and completing a search task in a novel DHI; they were encouraged to behave as if unobserved.

**Results:**

Lack of cultural specificity was highlighted as reducing relevance and usability, particularly relating to dietary change. Preferred features reflected individual health beliefs and behaviors, digital skills, and trust in DHIs. For example, tracking blood glucose was considered by some to be positive, while for others it caused distress and anxiety. Similarly, some users found the novel DHI to be extremely simple to set up and use, and others grew frustrated navigating through initial interfaces. Many participants raised concerns about data privacy and needing to agree to terms and conditions that they did not understand. Participants expressed that with information and support from trusted sources, they would be interested in using DHIs as part of self-management.

**Conclusions:**

DHIs may support South Asians to prevent and manage cardiometabolic disease, but it is important to consider the needs of specific user groups in DHI development, design, and implementation. Despite motivation to make health changes, digital barriers are common. Cultural appropriateness and trusted sources (such as health care providers and community organizations) have roles in increasing awareness and enabling individuals to access and use DHIs.

## Introduction

Use of digital health interventions (DHIs) for prevention and management of cardiometabolic diseases (CMD), such as diabetes, heart disease, and hypertension, has increased rapidly in the National Health Service (NHS) [1-3]. Digital health approaches, such as support with diet, activity, self-management, and remote monitoring, have shown some patient benefits, including reduction in cardiometabolic risk factors and outcomes [4,5], glycemic control [6], and reduced hospitalizations [7]. However, increasing use of DHIs may exclude some populations, particularly ethnic minorities at greater risk of health inequalities [8,9].

Although there is evidence related to improving accessibility of DHIs in African-American populations [10,11], there is limited evidence related to DHIs in South Asian populations in the United Kingdom [12,13], who face increased risk of CMD [14] and may be more likely to experience digital inequalities [15,16]. Previous research in South Asian populations in the United Kingdom considered acceptability and design issues related to SMS text messaging services [17]. Among South Asians, lack of awareness and the process of downloading and setting up DHIs have been identified as being specific barriers to ongoing engagement with digital health [18].

Approximately 25% of all apps are estimated to be uninstalled after only one use [19]. A meta-analysis of app use for chronic disease found that in real-world studies lasting between 2 weeks and 1 year, average attrition rate (negligible or ceased use) was 49% [20]. Understanding how participants react and interact with new DHIs and what features encourage them to explore further or stop use could inform recommendations for improvement. As more complex digital approaches (such as remote monitoring and virtual wards) are widely adopted, it is important to understand the needs of different population subgroups, such as South Asians. We aimed to understand the experience of DHI use by South Asian individuals with CMD, as well as how design and implementation can be improved to support uptake and use.

## Methods

### Ethical Considerations

Ethical approval was by the NHS London - Brent Research Ethics Committee (IRAS 261047). Informed consent was obtained from participants through a written consent form that was explained by the researcher. Participation was voluntary and participants could withdraw at any point of the study. There were no direct risks involved for participants. Data collected were deidentified and stored securely in accordance with the institution’s data management policies. Participants were reimbursed £50 (US $65.62) for their time.

### Think Aloud Interviews

A mixed (reactive and reflective) “think aloud” interview approach was used [21-23]. Interviews were conducted by MR and NK, who are both experienced qualitative researchers. Recruitment was via primary care, community organizations, and snowball methods, ensuring representation across South Asian ethnicity, age, geography, generation, and gender. Participants were English-speaking, had or were at risk of diabetes and/or heart disease (ie, prediabetes was included), and had access to a suitable device (smartphone, tablet, or laptop). Participants were offered WiFi access to facilitate downloading the app, if required.

After written informed consent, interviews lasting 45‐60 minutes were conducted by MR or NK in person, at a location convenient to participants. Participants were advised that the interview would be audiotaped, would be used for research purposes only, and would not be accessible to anyone outside the research team. It was stressed that the opinions of the participants were important, that there were no right or wrong answers, and that they could withdraw at any time. The interview guide is provided in Multimedia Appendix 1.

All participants were asked to use their device to navigate to the NHS website while explaining what they were doing as a warm-up think aloud activity. Participants who currently used a DHI were asked to navigate through it as usual, narrating what they were doing and why, for example updating and checking their step count.

All participants (including those who did not currently use a DHI) were asked to participate in the next part of the think aloud interview, involving an unfamiliar smartphone app chosen from a list provided by the research team. The 3 options were chosen from commonly used apps in a previous survey, which were in line with interview participant preferences for DHIs that were free, provided by a trusted organization (NHS), and available in iOS and Android online stores [18]. Participants were asked to choose 1 of these 3 apps ahead of the interview and to download it to their device. Support and WiFi were provided to those who needed to download the app during the interview.

Participants were asked to think aloud about the process of setting up these apps and, if they were able and willing to continue, to undertake one action on the app (eg, navigating to a specific page of the selected app). In the second half of the interview, participants were asked to describe and explain their thoughts and preferences on the use of DHIs to the interviewer, with the option of using their devices to demonstrate their views. Discussions focused on exploring use of technology as part of health, as well as barriers and facilitators to use. Participants were provided with a £50 (US $65.62) retail voucher as compensation for their time.

Recordings were professionally transcribed, and a reflexive thematic approach was taken to analysis [24]. Familiarization and initial coding was completed by MR and DRP, and merged for comparison. Through discussion between MR, DRP, NK, and DS, code definitions were revised, and transcripts further iteratively coded. These discussions drew on the team’s experiences in human-computer interactions, think aloud and qualitative methods, health inequalities, and long-term health conditions. Codes were initially grouped by MR, and themes were developed collaboratively through reflective notes and discussion between MR, DRP, DS, and NK.

## Results

### Participant Characteristics and Current App Use

A total of 18 participants with CMD were recruited, representing a range of gender, age, and ethnicity (Table 1). Most participants (n=11, 61%) currently used at least one DHI on their mobile phone, and a further 2 participants (11%) used their desktop, for example to access patient portals. Participants who did not use DHIs beyond receiving SMS text messages as part of appointment or other reminders were also included (n=5, 28%).

**Table 1.** Participant demographics.

	Participants, n (%)
**Ethnicity**	
Bangladeshi	5 (28)
Indian	7 (39)
Pakistani	6 (33)
**Gender**	
Female	8 (44)
Male	10 (56)
**Age range (years)**	
18‐34	1 (6)
35‐44	3 (17)
45‐54	5 (28)
55‐64	5 (28)
65‐74	3 (17)
≥75	1 (6)
**Education**	
Secondary	4 (22)
Tertiary	14 (78)
**Languages spoken**	
English	18 (100)
Bengali	4 (22)
Gujarati	4 (22)
Hindi	6 (33)
Punjabi	7 (39)
Urdu	6 (33)
Other/not provided	2 (11)
**Religion**	
No religious beliefs/none provided	2 (11)
Hinduism	4 (22)
Islam	10 (56)
Sikhism	2 (11)
**Location**	
Greater London	6 (33)
Midlands East	7 (39)
West Midlands	1 (6)
Yorkshire and Humber	4 (22)
**Health conditions**	
Prediabetes, type 1 diabetes, or type 2 diabetes	14 (78)
Coronary heart disease, hypertension	13 (72)

Overall, participants described their DHI use as relating to information seeking; supporting exercise and well-being; accessing resources recommended by health care professionals; booking appointments or checking test results; home monitoring (eg, for atrial fibrillation); and managing diabetes (continuous glucose monitoring [CGM]). Familiar DHIs demonstrated in the first think aloud task included the following: the NHS or NHS Covid app (n=3); wearable step counter (n=3, including one without an associated app); in-built mobile app used for step tracking (n=3); gym and fitness app (n=1); CGM app (n=1); heart rate monitoring app for atrial fibrillation (n=1); and no DHIs (n=6). Apps chosen in the second think aloud task (novel DHI) included Active 10 [25] (n=11); Weight loss [26] (n=4); and Couch to 5K [27] (n=2). One participant was not able to proceed with app download but participated in the interview.

The key themes identified included facilitators and barriers to use of digital tools for health; terms and conditions, permissions, and data privacy; the changing role of DHIs in addressing health needs over time; and personalization and adaptation to meet needs. Finally, participants offered recommendations for improvement of design and implementation.

### Facilitators and Barriers to Making Good Use Of Digital Tools for Health Management

All participants included in the study had a smartphone but had varying levels of engagement with digital health: “*…it’s not that we are not using [smartphones]. We are still using it but maybe not as freely as others would. If we could motivate ourselves a little bit more, maybe we could all make good use of it.”* [int 14, female, 53 years old]. Several common barriers to digital uptake were described by participants who had limited or no knowledge of digital health, including lack of digital skills, fear, and previous negative experiences such as scams, viruses, or errors leading to loss of money. Participants also spoke about barriers faced by others, including language, literacy, and digital access. Together these barriers had an impact on the ability of participants to interact with health services:

If they’re not answering the phone...they say “...make an appointment online.” But what am I supposed to say? I don’t know how to, you know, I haven’t got the app...So everything is made difficult for us.

[int 03, female, 49 years old, DHI nonuser]

Although some described themselves as not confident digitally, they were able to use an app if they had support in setting it up, such as in the place they receive care, or from family members or friends, they would be able to continue with app use independently. However, a few highlighted that while it was easier for family members to do it, then teach them, they were afraid about what would happen when their children left home, particularly as more services became digitized:

There’s something that as you’re getting older you think, this is digital…I’m like, God what can I do? Especially when the kids leave and everything, me and my husband we’re just going to be stuck…You know nobody’s reached out to us. So we’re just stuck where we are.

[int 03, female, 49 years old, limited use]

In the think aloud section related to setting up a novel (to participants) app, participants who considered themselves to be digitally confident found the process to be relatively easy and completed it swiftly. However, 5 of the 18 participants did not finish: 1 was unable to begin the task (“*I don’t know how to download other apps*.” [int 10, male, 71 years old, nonuser]) and 2 others did not have the digital skill to proceed. One participant told us: “*[I don’t know] the simplest sign like swipe to the left or right or do this*” [int 03, female, 49 years old], echoing other participants’ reflections on the lack of clarity or instructions about what to do when setting up or using an app for the first time.

Features that promoted engagement with the app included a simple interface, with clear presentation of available functions, and clear signposting and navigation. For example, one participant, who was using an affordable, wearable exercise tracker that did not have an app, praised the simplicity of the design and how it met her specific needs as she could “*keep pressing [a button to] look at the time and my steps and that’s it*” [int 03, female, 49 years old]. The inclusion of pictures and descriptions was praised as something that made it easy for anyone to operate, including those with language barriers. However, gaps were identified, including voice recognition not being good at picking up accented English, a lack of culturally specific information, and apps not always returning relevant results. For example, in relation to an app that was able to calculate calories based on a photo, one participant described its limitations:

You know if it’s just standard [Asian sweets] like gulab jamun…if you take a picture of that obviously you’ve got hundreds…of pictures of it on the internet but if it’s some other Asian dessert that’s not as common as that, the app might get a bit confused to what you’re eating.

[int 05, male, 40 years old, DHI nonuser]

Participants who were already using DHIs reported starting using them for a number of reasons, including the following: it was recommended by friends and family, some of whom also installed the app; a need to engage with new digital modes of contact with health care; a recommendation from their health care team as part of routine management; and increased confidence from app use in other areas (such as banking). Some participants described being willing to use DHIs, but they were not aware that they existed, needed more information about their function, or wanted recommendations from trusted sources such as the NHS.

For some, while they might use digital tools in other aspects of their life, limiting their digital use for health self-management was a choice. They expressed a preference to speak to another person directly (on the phone or face-to-face); found no benefit to app use over existing actions to manage their health condition; or considered current service provision to be satisfactory and did not need to use digital to engage with health care providers: *“…I can order [my prescriptions] as well through my app but I have not done it so far…usually they already given me [paper copy]…*” [int 18, male, 77 years old, DHI user]

Design and implementation features that participants highlighted as specific barriers to initial and continued use of digital for health purposes included the affordability of DHIs, complexity of sign-ins to maintain security, and suitability to manage their health needs. Affordability was raised in particular regarding CGM, which is available through the NHS for all adults with type 1 diabetes and for some adults with type 2 diabetes (eg, if insulin-treated or other clinical need is identified) [28]. The need to set up health apps in a secure fashion, such as remembering passwords, entering a lot of information, or setting up other security features, caused some people to pause the setup process to seek help or terminate the activity:

But it was quite difficult actually to go through the process [of setting up the NHS app to use the COVID passport]...they ask for the information but then they weren’t recognising like the face recognition...

[int 01, male, 52 years old, DHI user]

Linked to the reasons for stopping app setup, participants identified reasons that they might choose not to use the app. These include malfunctions (such as the app freezing or not syncing), needing to repeat log-in and administrative tasks, or a lack of integration with other apps—for example, participants might have to use multiple portals for appointment booking, reminders, and viewing test results. In addition, 2 participants described apps as having poor usability for individuals with complex needs (eg, cannot scroll and select all medications for reordering, or the buttons being too small on a smartphone, making entering data “tricky”), although in both these cases they opted to complete these tasks on their desktop computers rather than avoiding digital altogether.

### Terms and Conditions, Permissions, and Data Privacy: Participant Concerns About Agreeing to the Unknown

In the think aloud portion of the interview, many participants agreed to the terms and conditions without reading them or after taking only a cursory look, with one participant summarizing attitudes observed across the sample: “*Yes, I’m not going to view terms and conditions, but I will agree to them because nobody reads terms and conditions*” [int 08, female, 49 years old, DHI user]. A similar approach was taken in regard to permissions associated with the app (location and motion-tracking), with some participants choosing to accept to continue rapidly onto the app.

All participants were encouraged to stop installation at any point they wished, and of the 5 participants who did not complete installation of the app in the think aloud portion, 3 who did so had concerns about permissions and privacy. Difficulty with access to the terms and conditions through an external link; the length of terms and conditions and the technical language used; and not being sure of what they should be looking for were all highlighted as specific worries:

But I’m just wondering what- when it’s saying there’s a link, why can’t I find it, you know. If I put continue, it says I have to tick these two boxes. Because then you think “I might as well tick them”, don’t you?

[int 13, female, 64 years old, limited DHI user, did not continue with installation after this comment]

Participants did not necessarily understand the purpose and requirement for permissions. Some felt that they needed to agree to enable apps to work, for example in relation to tracking activity. Although generally attitudes toward the NHS and NHS-related DHIs were positive, 1 participant explained that for them, media coverage of issues around “track and trace” (the NHS COVID-19 contact tracing program) had undermined their trust in NHS data handling. Although the participant did not give further details, multiple instances of poor practices in relation to data handling were reported during the pandemic [29,30]. Unknowns about data sharing and privacy were raised in a number of different ways, around the purpose of sharing and the risk of potential misuse:

I mean my GP knows my medical history and my husband, but I don’t know if I want everybody to know it, you know. And how would they actually use it, you know. If I put [health details on the app]…how would they- what would they do with it? Is it necessary to put on there? I don’t know.

[int 13, female, 64 years old, limited DHI user]

A lack of clear and specific information about permissions led to concerns such as that agreeing to one set of permissions might give access to their data for another purpose: “*you don’t know what type of apps come to you with the main things but actually, tracking you for other things*” [int 06, male, 59 years old, DHI on PC only]. Another participant was worried about whether data from their devices could be linked externally:

[…] say I have [a smartwatch] I’m sending emails and like, [from its ID] you know that it’s me, right? So, then I’m sending my medical data and then it’ll be known that that’s my medical data. So, for somebody down the road, you know, i.e. in the database, it’ll be isolated as my data. That is concerning, yes. So, the anonymity isn’t there if I’m using that watch because linked to that watch is my, you know, account effectively.

[int 09, male, 55 years old, DHI user]

To mitigate these concerns, participants described being selective in the types of DHIs used (such as only for low-risk activities such as step tracking) or, in the case of 2 male participants, used multiple phones. However, they highlighted that there was a balance between concerns about privacy risks and perceived benefits, as 1 participant stated: “*But does [data sharing] really matter if you’re going to get a health benefit out of it?*” [int 09, male, 55 years old, DHI user].

### DHIs Play a Changing Role in Addressing Health Needs Over Time

Four main types of DHI benefits were described: accessing and keeping contact with health care providers (eg, appointment booking and reminders via SMS text messages, general practitioner platforms, or the NHS app); benefits related to COVID-19 during the pandemic (eg, information and vaccination passports); helping manage their overall health and well-being (eg, diet, exercise, and other lifestyle changes); and supporting the management of specific health conditions (eg, glucose, heart rate, or blood pressure monitoring). The discussions focused on DHIs for behavior change and specific support with health conditions, as well as the changing relevance of DHIs over time.

In relation to behavior change, participants identified that DHIs had supported their needs through features such as availability of relevant information, setting of manageable goals, tracking progress, motivation through rewards and competition with others, and prompting action through reminders and notifications.

I didn’t know this information, it’s quite helpful for me. I can walk the 10 minutes brisk walk…It’s good. And it’s the matter the information that people don’t know about these things and that is the information gap. If the people know these little information, these very tiny things, then many people can improve their health.

[int 12, male, 55 years old, DHI user, reflecting on the NHS app Active 10]

Yes, it encourages me because I do like to compare with my wife…who has done more. So it’s like a bit of a competition as well. So it’s- I find that really good, you know. At the end of the day, evening time I’ll see what I’ve done today. And then the next day if I’ve done less I’ll try and do more.

[int 01, male, 52 years old, reflecting on their step tracker]

General app features, such as heart rate monitors, were also considered meaningful to those with a family history of heart disease. Specific app features that were described as helpful included a personalized home screen with key information, clear visuals around progress and rewards (“your own personal win”), and reasonable prompts. For example, while demonstrating the in-built health app on their phone, 1 participant said:

…what I do here I go to the option “steps” to see what I have done. So I can see today I have only done 1,127 steps…but I can go for the weekly option there…and it also actually gives me the option to see my monthly data…I do [it] regularly, normally I actually see my weekly data and see what I have done.

[int 12, male, 55 years old]

However, most participants did not know where and how to find and select the apps relevant to their needs.

Although many participants described their experiences of digital health in relation to general engagement with the health system or behavior change for overall risk reduction, a few described them as part of a program of management of CMD. There were 2 participants who spoke about the use of CGM in diabetes, 1 for themselves, and another as part of managing their relative’s health. Specific benefits highlighted included real-time monitoring, ability to monitor family members remotely as part of a team with their caregivers, alerting to the need to rapidly respond, and improved understanding of how their glucose responded to their diet, which contributed to better long-term management of their diabetes.

I was actually able to keep my blood sugars at a constant because it become almost like, it’s like you gamify it you know…Very soon you learn what makes your sugar goes up and what makes it go down. And, it’s very sad to say this but the knowledge that I might go blind doesn’t make as much as a difference as the fact that I’ve got that little graph that’s telling me you’re going to hit nine.

[int 08, female, 49 years old, reflecting on experience of self-funded CGM]

It’s confidence, it’s less cost…You can imagine, you know, the ailments that you get from high sugar and low sugar. It was constant chaos and that’s kind of completely- almost completely taken that out. So, you’re in control, and you’re comfortable and relaxed about it really…

[int 09, male, 55 years old, supporting a family member with diabetes]

Additionally, changes to health over time could impact interactions with specific digital interventions. One participant described how they avoided looking at historical data as it reflected their decreased mobility: “*…it’s quite upsetting knowing that a couple of years ago I did nine, ten thousand steps and I can’t do it now*” [int 13, female, 64 years old, limited user]. Another highlighted how they preferred to make practical decisions based on their experience, rather than following technology that gave generic advice or was unable to adapt:

I think some people [become anxious and check] blood pressure like every day, two times a day…as long as I know I’m feeling alright a particular day, I don’t think I need to know, you know. My legs swell up a particular day…I just take it easy that day and elevate it as much as I can, you know support it. And I think I like to do it…in a more practical way. I don’t always want to be led by technology.

[int 13, female, 64 years old, limited user]

Perception of the accuracy of apps played a significant role in whether they considered them beneficial to their needs. In relation to activity trackers, a lack of explanation about how they work and how to set achievable goals meant that one participant discounted the readings as it would count when they moved their arm:

I don’t think they’re very accurate. So it’s like even if I’m sat there just moving my arm around and then I’ll try to get something, it just increases my tally of my steps I’ve done. So I know it’s not true.

[int 05, male, 40 years old, limited user]

Although some participants recognized the potential benefit of DHIs for others, they felt that they had limited benefit for themselves, for example, if they were already active (and did not need a reminder or tracker) or managed their health in other ways. There were 2 participants who expressed a preference for not taking a phone to track exercise due to concerns about theft or loss, although one used a simple wearable device instead.

Anxiety around health caused by searching for health information (“*it was like my addiction*”), constant monitoring, competing with others, not meeting goals, or potential inaccuracy of readings led to a few participants choosing to use their existing DHIs less or discontinue them:

…One reason I’m checking is because I want to know how I can make my sugar level go down. Other reason I don’t want to check is because when the sugar high, it makes me more anxious…We’re relying on all these numbers and digital and all the technology, and sometimes, of course it’s all the time is good, but sometimes we think, can we not just lead simple life? And sometimes I’m thinking that if machine is not working properly, then that’s giving us wrong reading and making you feel even more nervous.

[int 14, female, 53 years old, DHI nonuser]

Finally, the time-consuming nature of entering exercise or diet data, or reacting to notifications, was another reason that people did not feel DHIs could meet their current needs. A busy working mother, undergoing menopause, described an overload of notifications to her phone, which acted as an interface for her duties to other people: “*too many notifications to do- too many emails, too many phone calls to call back*.” However, while taking care of her health was an additional burden, she expressed an interest in trying: “*But I think maybe this…could be helpful for me at least to remind me to move. Maybe I- I will defer other notification…I need to make my health the priority, not always the other people*.”

### User Personalization and Adaptation of DHIs to Meet Their Needs

Participants described several ways in which they tailored their experience of DHIs to meet their needs. The first was in selection, trialing several apps before identifying the right one; for example, 1 participant with diabetes described the process of finding one for dietary management:

I think what has happened is over the past four, five years, I’ve tried to do different kinds of diet...it helps for an app to give you the information you require. So I tried [calorie counting app]...but I eventually ended up using [another] so yes, it’s been a trial and error thing. So yes, one after the other.

[int 08, female, 49 years old, DHI on PC only]

Participants also limited ways in which they engaged with apps to those that directly met their needs or were easy to use. As one participant put it: *“I want it in the most simplest form and whatever I think I need that’s what I’ve gone for.”* Participants who used in-built health apps for step tracking were aware of other features but did not consider them relevant at this time.

...I never actually use these things...There are many things to explore but to be honest this is it...I kind of focus on how many steps or how many miles I am doing.

[int 12, male, 55 years old]

This also varied temporally, such as choosing not to wear heart rate or activity monitors overnight due to discomfort, or using DHIs only when they felt they were needed. This need included changes in symptoms, or when they felt they needed to reestablish behavior change:

If my symptoms start telling me something I’ll check my blood sugar immediately and also if I’m feeling more thirsty, I’m going frequent to the toilet, I start noticing all these, check it on there whether I need to look and do something else.

[int 15, male, 69 years old, DHI user]

Although apps were considered useful for providing information, the actual practice of maintaining behavior change was identified as being challenging, with participants describing returning to previous behaviors after achieving change. Participants demonstrated a variety of “bargaining” strategies in how DHIs were used to manage health behaviors, such as not collecting data when the answer will be “bad” or only checking when they know it will be “good,” or using more than one app to compare data and predictions and using the preferred answer.

If you feel like you’re going off track and you’re suddenly going through sort of like a binge eating period, you can kind of see that there’s like a certain month period where I’ve not - I’ve just decided not to document what I’m eating because you don’t want to see that I’m having... [a] whole tub of [ice-cream].

[int 02, female, 42 years old]

For participants who had sufficiently embedded the required behavior change to achieve stable management of their cardiometabolic condition, DHIs were identified as having a natural endpoint or a reduced role:

There’s a certain amount of time, two or three months, that you kind of really use the [CGM] app and then after that it peters out because you’re comfortable...it’s almost the usefulness of the app is for that three months, right, and beyond that it’s almost like- almost like an insurance policy…

[int 09, male, 55 years old]

### Participant Recommendations for Improving Design and Implementation to Support DHI Uptake and Use

**Figure 1.**
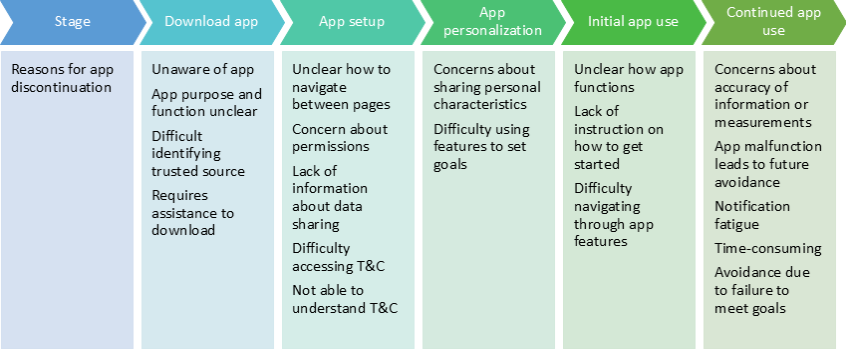
User pathways to app use for cardiometabolic disease and reasons for app discontinuation. T&C: terms and conditions.

Participants made several recommendations aimed at improving their own experience or that of those in their wider community that they thought could benefit from DHIs with some adaptations. Figure 1 summarizes the process of setting up and using a DHI described by participants, reasons for discontinuation, and opportunities to intervene to promote uptake and use of DHIs, using apps as an example.

Participants thought that the DHIs discussed had potential benefits for a wide range of people who were interested in making changes to their health. Suggestions for increasing accessibility in app features included: information provided in a range of languages, and with audio or video options; options for font size; visual aids on the page of where to click; and in-app support (such as live-chat options). They mentioned that relevance to the South Asian population could be improved through including a variety of cultural foods in diet-tracking apps, as well as including relevant celebrities, voices, or avatars in apps that included these. When introducing or implementing digital interventions, suggestions included the use of wearables or simple devices to promote exercise; installation and start-up support from health care providers; and providing safe spaces for digital upskilling and practice. Younger participants suggested utilizing social media such as WhatsApp and Facebook to engage people in issues around health as these were popular among the older generation who did have access to phones; however, there is mixed evidence supporting the use of social media for peer-to-peer information sharing [31] or as a channel for delivering diabetes education [32].

Participants also suggested engaging with the current “middle-aged” generation who could be supported to improve their health as they move into their fifties and sixties. In both app setup and ongoing use, participants wanted clear communication about the reasons for inputting personal details (ie, whether this information will be used to personalize the service and how this will be handled by third parties). Although goal setting was seen as beneficial, individuals suggested that they would benefit from instructions and guidance, such as in the form of a “how to” section or videos, as well as explaining exactly how the app works—for example, whether an activity-tracking DHI requires activation or collects information passively. The importance of keeping a range of options available was emphasized, including support for desktop versions of interfaces, and respecting the need to keep nondigital channels open for those with new concerns or for people who might choose to never use digital, including some older people.

In relation to their own use, participants wished for increased personalization and accountability, such as through digital programs that included coaching. Improved integration, particularly in accessing their health data, test results, appointments, and other NHS functions, was also seen as being beneficial. A participant who had not been offered CGM also identified that they would want something to “*do with my diabetes and I could see what my levels were. You know sometimes, because I have to do pricking every day*,” suggesting that there is an appetite for CGM among populations who are not aware of it or able to ask for it. Ideal DHIs included joined-up dietary support for those with diabetes, which would suggest and track food intake, and allow for inputting exercise details. Additionally, participants suggested the idea of a “lab on a watch,” which could act as a one-stop shop to monitor health.

The final areas of recommendations related to terms and conditions and data collection. Including a link to terms and conditions that navigated to a web page was confusing for some participants, and it would be helpful if it was integrated within the app. However, for a more meaningful way for a user to engage with the agreement, participants suggested that a lay summary of key messages should be included ahead of the checkboxes. Similarly, an explanation of the purpose of each permission, and how that data would be shared, would reassure people that the app was safe to use.

## Discussion

### Principal Findings

We present experiences of utilizing DHIs for prevention and management of CMD across a diverse group of UK individuals from a South Asian background, building on previously identified barriers and facilitators of digital acceptability, uptake, and use [18]. We highlighted willingness to use technology as part of prevention and management of CMD, but one single approach did not suit all, including the choice not to use DHIs among individuals who might otherwise use internet-based communication and entertainment, as described in other populations [33,34]. A review of nonuse of telemedicine also highlighted “other preferences” as a key aspect of attitudes toward telemedicine technologies, describing preferences for conventional solutions or other technical solutions [35].

Across all levels of digital skill, a key concern in downloading and setting up apps focused on data privacy and needing to agree to difficult-to-understand terms and conditions to use DHIs, including those for essential services. Recent studies in other contexts have also put forward ways to improve user engagement with terms and conditions, including not using hyperlinks and ensuring that they are transparently displayed early in user interaction [36]. Others have included terms of use as a factor to consider in a digital health evaluation tool for patients and clinicians [37]. However, many participants would not have been able to utilize this tool as it assumes a level of digital knowledge. There is a pressing need for improved communication and education for meaningful consent. Moreover, some concern about data safety within DHIs appears justified in light of evidence around vulnerabilities in medical devices and commercially available wearables [38-40], highlighting the need to provide assurance to the public.

The types of DHIs used by participants ranged from step counters to those with more complex features such as CGM. Features that were considered by one user to be positive (such as monitoring and tracking measurements) were reported by others to cause distress and anxiety. Regular DHI users found the NHS apps used in the “think aloud” portion of the interview to be extremely simple to set up and use, while participants who described themselves as less confident with technology grew frustrated navigating through the initial information screens. International recommendations on diabetes highlight the importance of designing apps with the level of technology proficiency of different patient populations in mind and increasing accessibility by using languages other than English, as well as increasing accessibility for people with visual impairment [41]. However, even when individuals did not face these barriers, a lack of cultural specificity in provided information reduced relevance and usability, particularly in relation to CMD, where dietary change may form a significant part of patient self-management [18,42].

Participants described dynamic changes in their interaction with and expectations of DHIs, reflecting variation in their health needs and exploration of approaches, in the short- and longer-term, both in relation to lifestyle change and management of specific CMDs. This supports previous findings in other populations such as those with diabetes, where after addressing initial acute needs after diagnosis, individuals may decrease use until a new event prompts interaction [43,44]; a similar pattern is demonstrated in people who may have a long-standing diagnosis but are newly introduced to DHIs. Digital approaches may also be seen as unsupportive or not relevant for newly diagnosed cardiometabolic conditions [18,45], which should be considered in South Asian populations as minority ethnic groups have been associated with an increased prevalence of diabetes distress [46].

### Comparison With Prior Work

Our findings in this study reflect in a health setting the framework for temporality of use of a new digital tool by Karapanos and colleagues: anticipation (participant expectations prior to use); orientation (excitement and frustration in learning and exploration); incorporation (meaningful use of product in daily lives); and identification (form a relationship with a product as it becomes part of their routine and social interactions) [47].

Positive impacts of DHIs for diabetes on individuals have been suggested to include helping them understand and feel in control of their condition; construct positive identities through being experts in their disease; increase their sense of power in clinician interactions; and demonstrate their goodness [44,48]. Although our findings reflect common positive outcomes in some users, we found that without adequate consideration of individual need, DHIs may instead increase anxiety or disempower patients. This may result in users feeling that they are being “led by the technology” rather than the technology supporting their needs, and this raises questions about the suitability and acceptability of automation in health interventions [49].

Studies in older populations in the United States have highlighted that people who are actively engaged in health management are more likely to use wearables and are willing to share health data with providers [50]. DHIs can be beneficial to older individuals when adapted for use and supported by communication with medical professionals [51,52]. Participants suggested a range of ways in which the design and implementation of digital technologies in health could support a diverse population, including promoting localization to different languages and cultures, including audio options; clear design and other accessibility measures; and provision of information and instruction within the app and through health care teams. Similar recommendations have been made for DHIs relating to CMD [53], in South Asian communities in the United Kingdom and India in relation to lifestyle change [42,54], for other health conditions [55-57], and for patient populations with different needs [58]. Embedding these approaches can have benefit beyond any single patient population [59]. However, for participants (of any age) who are digitally confident, more complex and integrated interventions may be suitable. This highlights the importance of having a range of interventions that are targeted to the needs and expectations of populations who may benefit from them (eg, other ethnic or minority groups) [60].

### Limitations

There is significant diversity within the South Asian population in the United Kingdom, by migration generation, country of origin, ethnicity, religion, education, occupation, and income, as well as by age and gender; with a relatively small sample size (n=18), this study cannot represent all experiences of digital access. In addition, due to the think aloud method, only participants with mobile phones or other devices able to support apps, and who were able to speak English, were recruited. In 2011 census data, most people of South Asian ethnicity in the United Kingdom were able to speak English, with nonspeakers tending to be older, female, and from Bangladeshi and Pakistani backgrounds [61]. This emphasizes the need for appropriate support to ensure these groups are able to access and benefit from health services for CMD. However, there is a significant difference between age groups; although Bangladeshi women over the age of 65 years reported the highest rate of not speaking English (44.9%), this was only 2.8% in the 25‐44 year age group [61]. This suggests that there is a large demographic of English-speaking South Asians in the United Kingdom that could potentially benefit from digital health approaches, given the right support.

There is currently limited evidence on the experiences of digital health among South Asian populations in the United Kingdom, particularly in relation to CMD [12,13]. This study makes an important contribution toward understanding opportunities for culturally relevant DHI design and implementation. In addition, broader findings and recommendations have the potential to benefit other populations that may currently be digitally excluded. Future research should explore implementation approaches to support those new to digital to install and use apps, as well as explore how to improve communication around permissions, privacy, and terms and conditions to ensure patients can meaningfully consent to the use of DHIs.

### Conclusion

In our study, we demonstrated that individuals from a South Asian background in the United Kingdom are interested in DHIs as part of prevention and management of CMD, and in addressing short- and long-term needs, including engaging with family members and carers for the benefit of those who may be otherwise digitally excluded. Initial access to technology (by which we mean awareness, downloading, and setting up a device or app) is a significant barrier. This emphasizes the importance of support from appropriate trusted sources, such as health care teams, in initiating DHI use. Suggestions to improve relevance and utility of DHIs for the South Asian population in the United Kingdom focused on inclusion of cultural foods in advice around dietary management of CMD. Participants made a number of recommendations around DHI design and implementation that would improve accessibility across user groups. This highlights that design approaches for DHIs for prevention or management of cardiometabolic disease should take into account the diverse needs of many populations for universal benefit.

## Supplementary material

10.2196/57338 Multimedia Appendix 1 Interview guide.

## Abbreviations

CGM: continuous glucose monitoring
CMD: cardiometabolic disease
DHI: digital health intervention
NHS: National Health Service
