# Supplementary material for: Uptake of Digital Health Interventions for Cardiometabolic Disease in British South Asian Individuals: Think Aloud Study
Source: JMIR Hum Factors. 2024 Oct 24;11:e57338. doi: 10.2196/57338 (PMC11526767; doi:10.2196/57338)
Supplement: Multimedia Appendix 1 [file humanfactors-v11-e57338-s001.docx]

**Appendix 1: Interview guide**

*This guide sets out an indicative list of questions and prompts to guide the interview through the key themes of interest, based on DHI use of the participant.*

| Study and participant introduction | - Welcome - Permission to audio record - Introductions - Introduction of interview aims and structure - Allow time for questions before start |
| --- | --- |
| Introductory questions | *Warm up introductory questions* |
|  | Current DHI users   - (Confirmation) Which DHI do you use? - How often do you use the DHI? - When did you last use it?   Never users   - (Confirmation) Have you ever used a DHI to manage your heart disease or diabetes? - Have you used a DHI for other health conditions, or to stay fit? For example, an exercise or diet tracker?   Discontinued users   - (Confirmation) Which DHI did you use? - How long did you use it for? - When did you last use it? |
| *Introduction to ‘think aloud’* | *Introduction of ‘think aloud’ interview type and practice exercise* |
|  | In the first part of this interview I am going to ask you to use some websites or apps on your device, and to simply say out loud what you are thinking or doing, as you are doing it. You can say whatever comes to your mind as you do this – this could be what you’re looking at, thinking or doing. It may feel a bit awkward but I’ll remind you to keep talking if you forget. I will sit beside you and watch what you are doing on your screen. After we do this, we will have time to talk further about your experience of using the DHI.  We will start with a small task to practice speaking aloud. Please use your device to visit the NHS website. The address is [www.nhs.uk](http://www.nhs.uk). While you are doing this, try to say everything that goes through your mind.  *Prompt: Please keep on talking.*  Do you have any questions before we move onto the next thing? |
| *Part 1* | *Current app users* |
|  | Now we will move on to the app that you have said you are currently using [name].  Please use the app as you normally would. While you use the app please say aloud what you are thinking or doing.  Thank you. Are there other things that you do on the app less often? Can you go to that area of the app, and say what you are doing?   - *Why do you use these parts less often?*   What do you like about the app? Can you show me?   - *Why do you like this part/feature of the app?*   Are there any parts of the app that you don’t use, or don’t like to use? Can you show me? |
| *Part 2* | *All participants* |
|  | Please [download app they have not used before, or go to web-based tool].  SET UP activity/if easy choose one task  I would like you to spend the next few minutes using this app. Please continue to say out loud your thoughts and what you are doing.   - *Design one task based on app/web-based tool chosen (e.g. find information on diet, input exercise)*   What do you like about this app?  - How easy is it to use?  - Does it address your needs (e.g., for cardiovascular disease or diabetes)  - Would you carry on using it in the long-term?  Have you had problems using it? Can you show me? |
| *Part 3* | Semi-structured interviews |
|  | **All participants**  Use key points raised during the ‘Think aloud’ portion to cover positive and negative experiences of usability and content. Sample questions:   - When you were using the DHI you [*had the following reaction to use or content of the DHI*]. Can you tell me more about why you think this? - When you were using the DHI you mentioned [*errors/problems*]. Can you tell me more about this, and if there are any changes that would help? - You described [*positive experience*]. Can you tell me why you found this useful?   **Participants who currently use DHIs**   - Can you tell us more about your experience of using this DHI to help you manage your health condition? - Have you needed or received support or information to use this DHI? - How easy is this DHI to use? What about it makes it easy or difficult to use? - What do you like most/least about this DHI? - Have you used other DHIs for health before? How does this compare? - Are there any extra features of this DHI that you find useful? [e.g. additional information or ‘top tips’ sections] - Do you think there are any specific changes that would make it more helpful or easier to use for South Asians or for other groups of people? - Have there been any changes to the way you use DHIs during the coronavirus pandemic?   **Participants who discontinued use of DHIs**   - Can you tell us more about your experience of using this DHI to help you manage your health condition? - Did you need or receive support or information to use this DHI? - What was the reason you stopped using this DHI? - Were there any features that you found positive? - What changes would have encouraged you to keep using the app? - Have you used other DHIs for health before? What were the reasons for using/not using them? - Do you think there are any specific features that would make apps more helpful or easier to use for South Asians or for other groups of people? - Have there been any changes to the way you use DHIs during the coronavirus pandemic?   **Participants who have never used DHIs**   - Can you tell us why you have not used a DHI to help you manage your health condition? - Would you be interested in using DHI in future?   - What is the reason you would/not be interested in using a DHI in future?   - What support or information would help you to use a DHI? - At the start of the interview you said you had/had not used other DHIs for health:   - What were the reasons for using/not using them?   - Can you tell me more about your experience of using this DHI? - Do you think there are any specific features that would make apps more helpful or easier to use for South Asians or for other groups of people? - Have there been any changes to the way you use DHIs during the coronavirus pandemic? |
| Wrap-up | - Summarise key points in each topic - Any new or closing comments from participants |
| Session close | - Thank for participation - Signpost to consent form regarding consent, data protection, outputs of session and contacting research team - Remind participants that they will receive their £50 voucher and a sources of further support sheet. |
